# Michigan State Dental Society

**Published:** 1887-05

**Authors:** 


### PROCEEDINGS OF SOCIETIES.

### Discussion of Papers, Michigan State Dental Society.

### DISCUSSION ON DR. DORRENCE'S PAPER.

*Dr. Harroun.*—I wish to say a few words in reference to Dr. Mellotte and his material. The Executive Committee wished me to write to several persons, and also to any others whom I thought proper to come here. I wrote to Dr. Mellotte and told him to come here and demonstrate his system of Bridge and Crown Work. I received the statement. It was impossible for him to do so for he was in California. I told him I did not feel justified in demonstrating his material, but that I would do what I could to get it introduced. The material is very good. It will take a good impression, and quickly.

*Dr. Dorrance.*—The surface may be made free from stickiness by the use of French chalk. This is one of the impression materials that is easily changed in shape. The teeth should be dry. You must carefully remove the surplus.

*Dr. Douglass.*—I would like to suggest one thing more in addition to what Dr. Dorrance has said. I have many times adopted the plan of chilling the impression before taking from the mouth. In a few minutes you can chill the wax so that you have to use considerable force to remove it from the mouth when there are teeth present. You will find this very good. In using Modeling Composition be careful not to get it too cool before removing.

If it is not out of the way, I would like to ask for information. It is where we wish to take an impression of the mouth to make a lower set of teeth. The teeth have been out a long time, and the gums have receded, and the portion of the gums that the teeth set in are fleshy double over. The portion of the alveolus has absorbed. How are you going to make a set of teeth that will fit the mouth?

*Dr. Land.*—MR. PRESIDENT: This seems to be a very decided argument in favor of Modeling Compounds and a very strong one against plaster. I will be very sorry to give up plaster and go back to wax. I have done almost all kinds of work, and I am fully convinced that we have only one substance with which to take an impression, and that is plaster of Paris. It is the only substance that we can undercut. It is impossible to use Modeling Compounds for that. If we take plaster in its proper condition any impression can be taken, and that perfectly. In regard to using these componnds over I think if I should tell my patients that, they would not come back again.

*Dr. Robinson.*—MR. PRESIDENT: It is almost presumptious to go to the seat of learning to differ with the gentleman who has given this lecture. I have always been in favor of plaster impressions, but perhaps I do not know all, I would not be surprised if I did'nt. I do not know, but that it is best to use Modeling Compounds, but I must say it is hard to teach old dogs new tricks.

*Dr. Dorrance.*—MR. PRESIDENT: Before answering Doctor Douglass' question, I would like to remove the impression that has been entertained by some present. I am not here, an apostle of Modeling Compounds, but to show the use of Compounds, and I think if all knew how to use them they would like them. Where there is a flabbiness of gum I should cut it away, the patient permitting, and let it heal.

*Dr. Scott.*—MR. PRESIDENT: It occured to me that perhaps I have been set against the use Compositions in the use of plaster and wax. I find in my practice that it has been convenient to take a small quantity of beeswax and warm it, placing it in the impression cap just where you wish it, then quickly prepare your plaster, and press that in, and I think in many instances we get a very good impression, and readily remove it, where we could not do so if it were entirely of plaster.

*Dr. Taft.*—I should like to emphasize the remarks upon cleanliness in the matter of taking impressions. This is a matter that ought to receive consideration in practice, at least that everything that should render the operation easy and the most acceptable to the patient, should receive attention. In taking impressions, the patient should have every possible consideration; it is unpleasant at best, and so far as possible every thing that would annoy the patient should be avoided, have every thing scrupulously clean. In securing successful impressions of the mouth, much depends upon the bearing and manner of the operator.

The idea of using the material over and over again would be repulsive to any person of refined taste. Be careful that the mouth be not too much filled up, just material enough and no more. Escape of saliva should be avoided. Clean napkins should be about the person. Have the patient in the most quiet state, then the best results will be obtained.

[*To be continued.*]

